# Conventional and Unconventional Mechanisms by which Exocytosis Proteins Oversee β-cell Function and Protection

**DOI:** 10.3390/ijms22041833

**Published:** 2021-02-12

**Authors:** Diti Chatterjee Bhowmick, Miwon Ahn, Eunjin Oh, Rajakrishnan Veluthakal, Debbie C. Thurmond

**Affiliations:** Department of Molecular and Cellular Endocrinology, Beckman Research Institute of City of Hope, Duarte, CA 91010, USA; dcbhowmick@coh.org (D.C.B.); mahn@coh.org (M.A.); euoh@coh.org (E.O.); rveluthakal@coh.org (R.V.)

**Keywords:** insulin secretion, exocytosis, STX4, DOC2b, β-cell mass, β-cell function, β-cell senescence/aging

## Abstract

Type 2 diabetes (T2D) is one of the prominent causes of morbidity and mortality in the United States and beyond, reaching global pandemic proportions. One hallmark of T2D is dysfunctional glucose-stimulated insulin secretion from the pancreatic β-cell. Insulin is secreted via the recruitment of insulin secretory granules to the plasma membrane, where the soluble N-ethylmaleimide-sensitive factor attachment protein receptors (SNAREs) and SNARE regulators work together to dock the secretory granules and release insulin into the circulation. SNARE proteins and their regulators include the Syntaxins, SNAPs, Sec1/Munc18, VAMPs, and double C2-domain proteins. Recent studies using genomics, proteomics, and biochemical approaches have linked deficiencies of exocytosis proteins with the onset and progression of T2D. Promising results are also emerging wherein restoration or enhancement of certain exocytosis proteins to β-cells improves whole-body glucose homeostasis, enhances β-cell function, and surprisingly, protection of β-cell mass. Intriguingly, overexpression and knockout studies have revealed novel functions of certain exocytosis proteins, like Syntaxin 4, suggesting that exocytosis proteins can impact a variety of pathways, including inflammatory signaling and aging. In this review, we present the conventional and unconventional functions of β-cell exocytosis proteins in normal physiology and T2D and describe how these insights might improve clinical care for T2D.

## 1. Introduction

Diabetes mellitus is a complex and heterogeneous disease characterized by progressive loss of function in the insulin-secreting pancreatic β-cells. Diabetes can be largely classified into type 1 (T1D) and type 2 (T2D), with worldwide occurrence rates of ~5% and ~95%, respectively [1]. Based on the 2020 Standards of Medical Care in Diabetes from the American Diabetes Association, T1D involves autoimmune β-cell destruction, whereas T2D is characterized by progressive loss of β-cell insulin secretion frequently on the background of insulin resistance [2]. Hence, although the pathogenesis of T1D and T2D is mediated by immune vs. metabolic stress, respectively, the common outcome of both T1D and T2D is the loss of functional β-cell mass and resulting hyperglycemia.

Histological analysis of the pancreatic islets from T2D human donors shows a 40% average reduction in the β-cell mass (range 25–60%), increased amyloid deposits and β-cell apoptosis, reduced insulin content, and unaltered α-cell mass as compared with non-diabetic control pancreas samples [3,4,5,6,7,8,9]. Interestingly, although multiple studies have reported a substantial reduction in β-cell function (~80%) at the onset of T2D [8,10,11], this early loss of function is not coupled with the dramatic loss of β-cell mass that has been observed later in patients with a longer disease history [6,8]. Thus, β-cell dysfunction without β-cell loss is thought to be an early player in T2D pathogenesis.

T2D is associated with considerable morbidity and a significant decrease in lifespan. Several therapies are available for T2D treatment, such as metformin, metformin plus GLP-1 analogs, insulin plus metformin, and pioglitazone. However, none has been successful in preventing β-cell dysfunction and demise over time (reviewed in [1,12]). Hence, alternative therapeutic approaches to preserve or restore β-cell function remain highly sought-after.

There is growing recognition for the importance of exocytosis proteins, such as soluble N-ethylmaleimide-sensitive factor attachment protein receptors (SNAREs) and their regulators, in improving β-cell insulin secretion and peripheral insulin-stimulated glucose uptake. The importance of exocytosis proteins has also been demonstrated in other diseases, such as cancer and neuronal disorders [1,13,14,15,16,17], providing clues that these proteins could be drug targets. More recently, the beneficial role of the SNARE protein Syntaxin 4 (STX4) in promoting whole-body glucose homeostasis, healthspan, and longevity has been unveiled [18,19,20]. In this review, we highlight current advances that have clarified the role of exocytosis proteins in preserving β-cell health and function, with a particular focus on the SNARE protein STX4.

## 2. Exocytosis Proteins and β-Cell Function

### 2.1. Introduction to Insulin Secretion

**Figure 1:**
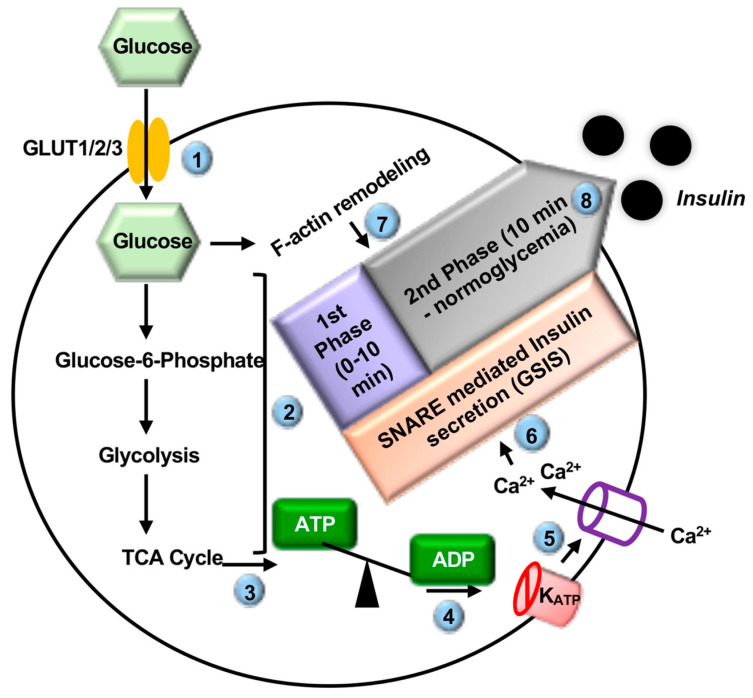
The steps of glucose stimulated insulin secretion (GSIS). Glucose enters the pancreatic β-cell via the GLUT1 or 3 (human)/GLUT2 (rodent) transporter (❶) and is rapidly metabolized via glycolysis and the tricarboxylic acid (TCA) cycle (❷). This increases the ATP/ADP ratio (❸), thereby closing the plasma membrane (PM)-localized ATP sensitive potassium channels (K_ATP_) (❹), resulting in depolarization of the PM, opening of voltage-sensitive Ca^2+^ channels at the PM (❺), and influx of Ca^2+^ into the β-cell. Increased Ca^2+^ (❻), as well as glucose-induced F-actin remodeling (❼), results in soluble N-ethylmaleimide-sensitive factor attachment protein receptor (SNARE)-mediated fusion of insulin secretory granules to the PM and biphasic insulin release from the β-cell (❽). Steps are indicated by (circled numbers).

Insulin is a peptide hormone biosynthesized and secreted from the pancreatic β-cell and is the master regulator of glucose homeostasis. Glucose-stimulated insulin secretion (GSIS) refers to the exocytosis of insulin from the insulin secretory granules (ISGs) of the pancreatic β-cell in response to elevated blood glucose concentrations (Figure 1). Extracellular glucose enters the β-cell via the plasma membrane (PM)-localized GLUT1/2/3 transporter proteins (GLUT2 in rodents, GLUT1/3 in humans) [21,22,23,24]. Once intracellular, glucose is rapidly metabolized, thereby increasing the ATP/ADP ratio. This results in the closing of PM-localized ATP-sensitive potassium channels (K_ATP_), depolarization of the PM, opening of voltage-sensitive Ca^2+^ channels at the PM, and influx of Ca^2+^ into the β-cell [25,26]. Elevated intracellular Ca^2+^ leads to SNARE-mediated ISG-PM priming and fusion, the formation of the fusion pore, and the release of insulin cargo extracellularly into the circulation [27,28].

Both in vitro and in vivo experiments have demonstrated that insulin is released from the β-cell in a pulsatile fashion, unlike the continuous release that is characteristic of other endocrine cells [29,30,31]. Early electron microscopy studies revealed that a small portion of the ISG pool is located within 100–200 nm of the β-cell PM, while the rest of the ISGs are located distal to the PM (reviewed in ref [32]). Based on this observation and similar knowledge from observations of neurotransmitter release from neurons, it was hypothesized that the membrane-proximal pre-docked ISGs constitute a readily releasable pool (RRP) of ISGs, which are released as the first response to glucose stimulation (reviewed in ref [32]). The distal storage pool of ISGs was similarly hypothesized to remain in the cytoplasm of the β-cell and await recruitment to the PM during depletion of the RRP (reviewed in [33]). This concept fits well with the observation that GSIS is biphasic. The first phase of GSIS is a transient insulin spike, lasting ~10 min, coinciding with the release of the RRP [34,35,36]. A sustained second phase of GSIS then ensues and can continue for hours until normal blood glucose levels are restored [37,38]. This second phase of GSIS requires the recruitment of the distal pool of ISGs (including newcomer ISGs), a process requiring the dynamic reorganization of the actin cytoskeleton [39].

ISG exocytosis is tightly regulated by the SNAREs, which are highly conserved proteins that closely resemble the vesicle fusion mechanism in neurons as well as exocrine, hematopoietic, and endocrine cells. SNARE proteins involved in ISG exocytosis are primarily categorized into two types: (i) Vesicle or “v”-SNAREs on the ISGs and (ii) target or “t”-SNAREs on the PM. Both v-SNARE and t-SNARE proteins bring the vesicle and target membranes (PM in this case) into proximity for fusion. Ultrastructural studies of the SNARE complex have revealed that one v-SNARE (VAMP2 in the β-cell) binds with two cognate t-SNARE proteins ((Syntaxin 1/4 (STX1/STX4) and SNAP23/25 in the β-cell)) in a heterotrimeric 1:1:1 ratio to form the SNARE core complex [40,41,42,43,44,45], which ultimately leads to membrane fusion and insulin release from the β-cell.

### 2.2. Role of Syntaxin Proteins in SNARE-Mediated Insulin Secretion

**Figure 2:**
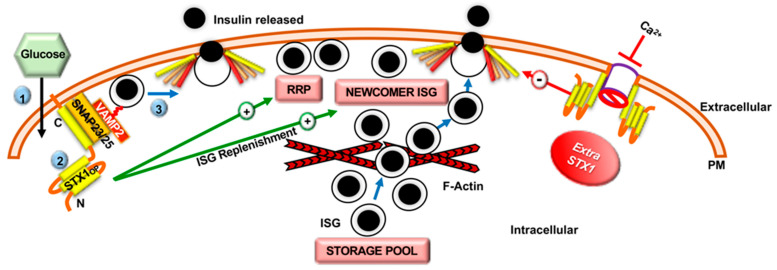
Role of STX1 in regulating insulin secretory granule (ISG) Pools and GSIS. During first–phase GSIS (0–10 min following glucose entry) (❶), three SNARE proteins: The STX1 open form (STX1_OP_), SNAP23/25, and VAMP2, assemble to form a heterotrimeric SNARE complex (❷) in the β-cell. This leads to fusion of the ISGs with the plasma membrane and release of insulin cargo into the extracellular space (❸). STX1 positively regulates the readily releasable pool (RRP) and newcomer pool of ISGs in the β-cell. Overexpression of STX1 decreases Ca^2+^ channel activity by physically interacting with it and thereby reduces GSIS. Blue arrows depict ISG movements, “+” arrows depict positive regulatory roles, “-“ arrow depicts a negative regulatory role. Steps are indicated by (circled numbers).

The importance of STX proteins in GSIS was first revealed by the study using anti-STX1 antibodies and global STX1 knockout (KO) mice, which showed a requirement for STX1 in insulin exocytosis and docking of the ISG RRP [46]. In line with these observations, a recent study using β-cell-specific STX1 KO (β-STX1 KO) mice revealed a new role for STX1 in both ISG exocytosis and replenishment. The β-STX1 KO mice showed impaired first- and second-phase GSIS, linked to severe reductions in the ISG RRP, as well as reduced recruitment of the distal ISG pool [47] (Figure 2). Intriguingly, in disagreement with the knowledge gathered from STX1 knockout (KO) mice in the context of the β-cell function, mice overexpressing STX1 in the islet β-cells showed decreased insulin exocytosis and perturbed activity of L-type Ca^2+^ channels, contributing to whole-body glucose intolerance [48]. These cumulative results indicate that STX1 has a narrow window of efficacy in the β-cell, wherein too little STX1 impairs GSIS, and too much STX1 causes novel interactions that impair β-cell function (Figure 2). STX1 overexpression in neurosecretory cells has a similar effect, indicating that the effects of excess STX1 on cellular function are similar across secretory cell types [49]. Interestingly, an early study demonstrated that cleavage of STX1 by botulinum toxin only reduced β-cell GSIS by 25% [50]. This result indicated that a STX1-independent insulin secretory complex must be present in the β-cells, possibly comprised of alternate STX isoforms.

**Figure 3:**
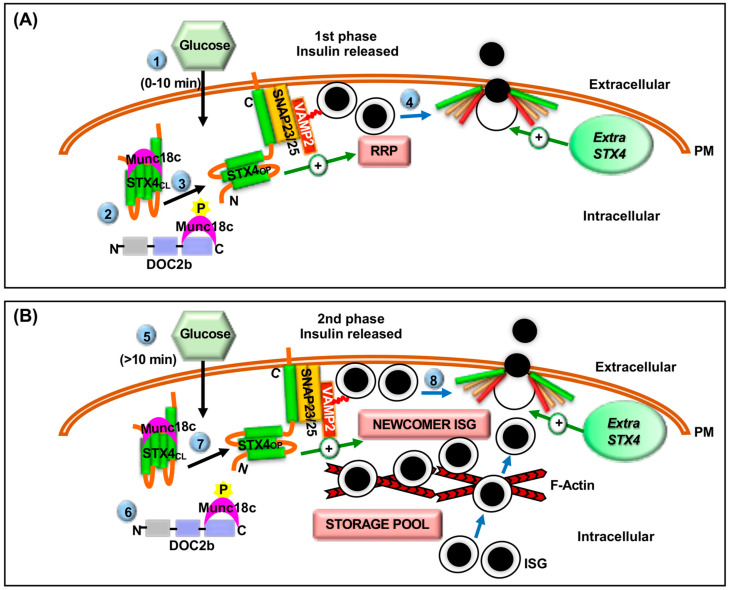
Roles of Syntaxin 4 in regulating ISG Pools and GSIS. (**A**) During the first (glucose stimulation 0–10min) (❶) and (**B**) second phases (glucose stimulation >10 min) (❺) of GSIS, (A, B) glucose stimulation tyrosine phosphorylates Munc18c and tyrosine phosphorylated Munc18c transiently switches its binding from STX4 to DOC2b (❷, ❻) in the β-cell. This results in the transition of STX4 from its closed (STX4_CL_) to open (STX4_OP_) conformation and the assembly of the three SNARE proteins to form a heterotrimeric complex: STX4, SNAP25 or SNAP23 and VAMP2 (❸, ❼) in the β-cell. This leads to SNARE-mediated ISG fusion with the PM and release of insulin cargo into the extracellular space (❹, ❽). STX4 positively regulates the RRP of ISGs. STX4 also positively regulates ISG refilling or newcomer pool of ISGs, possibly via its direct interaction with F-actin in the β-cell. Extra STX4 increases the amplitude of both phases of GSIS and enhances glucose homeostasis in vivo. Blue arrows depict ISG movements, “+” arrows depict positive regulatory roles. Steps are indicated by (circled numbers).

Indeed, the pancreatic β-cell expresses four PM-localized STX isoforms: STX1, STX2, STX3, and STX4 (reviewed in [1,32]). The importance of STX4 was revealed using global heterozygous KO of STX4 (−/+) in mice, which reduced first-phase GSIS by 50% and was fully rescued by overexpression of recombinant STX4 [51]. In contrast to the glucose intolerance observed in STX1A-overexpressing mice, STX4-transgenic mice with 2–5-fold overexpression of STX4 in the skeletal muscle, adipose tissue, and pancreatic tissues, showed improved glucose homeostasis and islet function [52]. Indeed, as little as a 2-fold increase in islet STX4 expression in the STX4-transgenic mice increased insulin secretion by ~35% during both phases of GSIS (Figure 3), indicating that STX4 positively regulates both phases of GSIS [51]. Consistent with these animal studies, shRNA mediated knockdown (KD) of endogenous STX4 in primary human islets followed by determination of GSIS by islet perifusion assay showed a reduction in both phases of GSIS by ~40–42% [53], supporting the existence of a key role for STX4 in GSIS in both mice and humans. Further analyses of single ISG behavior using patch-clamp capacitance measurements and total internal reflection fluorescence microscopy revealed that STX4-KD β-cells are characterized by severe loss of the RRP and impaired mobilization of ISGs to the inner PM surface, potentially explaining the loss of both phases of GSIS [53]. Interestingly, the authors also reported a concomitant reduction in the exocytotic protein Munc18c (46%), but not other exocytotic proteins, in the STX4-KD human islets (77% KD), which again highlighted the various roles of STX4 in regulating both phases of insulin secretion [53]. This same dependency of Munc18c level relative to STX4 alteration was also seen in mouse models of STX4 KO or overexpression [54,55]. Marked reduction in STX4 protein in the T2D donor islets (~70% reduction) and significant improvement of GSIS in otherwise secretion-deficient T2D islets following STX4 replenishment holds the key to the therapeutic potential of STX4 in diabetes treatment [20].

In addition to STX1 and STX4, STX3 participates in second-phase GSIS via interacting with the R-type/Cav2.3 Ca^2+^ channel α1 subunit and regulates exocytosis of newcomer ISGs [56,57]. In contrast to STX1, STX3, and STX4, STX2 is known to be an inhibitory SNARE protein in mammals as global STX2 KO mice have enhanced recruitment of newcomer ISGs in their β-cells that enhances GSIS [58]. Taken together, these results provide evidence for the important but non-identical roles of the four PM-localized STX isoforms in the β-cells that collectively regulate insulin secretion contributing to the maintenance of glucose homeostasis.

### 2.3. Role of SNARE-Associated Proteins in Insulin Secretion

The SNARE-associated proteins Sec1/Munc18 and double C2-domain-containing protein (DOC2) assist in the assembly and formation of the core SNARE complex (reviewed in [1,32]). Understanding the molecular regulators of insulin exocytosis can drive therapeutic discovery by clarifying which molecules are critical for each step in the pathway.

Sec1/Munc18 has three isoforms: Munc18a (also called Munc18-1), Munc18b, and Munc18c [59,60], which interact with specific PM-localized STX proteins to regulate exocytosis. All three Munc18 isoforms are expressed in the islet β-cell [51,54,61]. Munc18a and Munc18b bind to STX1, STX2, and STX3, whereas Munc18c only interacts with and regulates STX4 [62,63,64,65]. In the β-cell, Munc18a regulates pre-docked ISG fusion via its interaction with STX1-SNAP25-VAMP2 and thereby influences first-phase GSIS [66]. Recently, STX1 was shown to be recruited to the ISG docking site in a Munc18a-bound conformation in rat clonal β-cells, which provides the mechanical insight for the requirement of both the STX1 and Munc18a for granule docking in the β-cell [67]. In line with these, reduced Munc18a level in the T2D islets depicts the contributory role of Munc18a in the development of β-cell dysfunction during diabetes [20]. Similar to Munc18a, Munc18b has also been shown to regulate SNARE complexes. However, unlike Munc18a, Munc18b appears to regulate STX3-based SNARE complexes, and via interaction with VAMP8-based newcomer ISGs in particular [68,69]. These distinct SNARE complex and ISG population binding and regulatory specificities of the Munc18 family of proteins depict extraordinarily exquisite control metering ISG exocytosis in pancreatic β-cells [69].

The importance of Munc18c was revealed using pancreatic islets isolated from Munc18c (−/+) heterozygous KO mice and RNAi-mediated KD of endogenous Munc18c in a mouse clonal β-cell line. These Munc18c-deficient cells demonstrated selective deficits in second-phase GSIS and decreased STX4 accessibility to VAMP2 [59]. Also, electron microscopy revealed fewer ISGs juxtaposed to the PM in the Munc18c-depleted cells, indicating a functional requirement for Munc18c to mobilize ISGs during GSIS [59]. Consistent with the studies using rodent β-cells, studies using dispersed primary human islets with lentivirus-mediated knockdown of endogenous Munc18c confirmed Munc18c’s involvement in the exocytosis of predocked and newcomer ISG pools, as well as its requirement for both the first and second phases of GSIS [60]. However, Munc18c is not found in the SNARE complex in β-cells per se. Instead, in response to glucose, Munc18c becomes tyrosine-phosphorylated and transiently dissociates from STX4, presumably transitioning STX4 from its closed to open and activated conformation, thereby indirectly facilitating SNARE complex formation [70,71] (Figure 3). Munc18c-STX4 complexes are further regulated by the competitive binding of DOC2b to Munc18c, whereby phosphorylation of Munc18c at tyrosine 219 acts as a molecular switch to reduce its binding affinity for STX4 and enhance binding to DOC2b [71] (Figure 3). Taken together, increasing evidence points towards the crucial functional contribution of different isoforms of Munc18 proteins in regulating β-cell function. This is again reinforced by the fact that while most of the non-β-cells express either one or two dominant Munc18 isoforms, the pancreatic β-cell expresses all three Munc18 isoforms and each of them contributes towards the regulation of GSIS in an overlapping yet independent manner.

DOC2 has two main isoforms, DOC2a and DOC2b; DOC2a is primarily expressed in the pancreatic islets and neurons and DOC2b is ubiquitously expressed [72,73,74]. Islets from DOC2a KO mice have normal islet function [75], but DOC2b KO mouse islets have defects in both phases of GSIS [75,76]. Moreover, transgenic mice with DOC2b overexpression in the pancreas, skeletal muscle, and adipose tissue showed improved whole-body glucose tolerance and peripheral insulin sensitivity, and isolated islets showed enhanced GSIS [77]. Accordingly, inducible β-cell-specific DOC2b overexpressing transgenic mice exhibited improved whole-body glucose tolerance and enhanced islet GSIS, as well as resistance to the diabetogenic stimulus multiple-low-dose streptozotocin (STZ), which causes loss of β-cell mass and glucose intolerance [78]. DOC2b is a 46–50 kDa protein comprised of an N-terminal Munc13-interacting domain (MID) and C-terminal tandem Ca^2+^ and phospholipid-binding C2 domains (C2A and C2B). DOC2b interacts with Munc18a and Munc18c via its C2A and C2B domains, respectively [79], and appears to serve as a scaffolding platform for transient binding of Sec1/Munc18 proteins to facilitate SNARE complex formation and GSIS [79] (Figure 3). However, it remains unclear whether this scaffolding mechanism, presumed to impact ISG exocytosis and hence β-cell function, also underlies DOC2b’s ability to protect β-cell mass. Indeed, since β-cell dysfunction is now recognized as a precursor to loss of β-cell mass, this remains an intriguing possibility.

### 2.4. F-Actin Remodeling in Insulin Secretion

**Figure 4:**
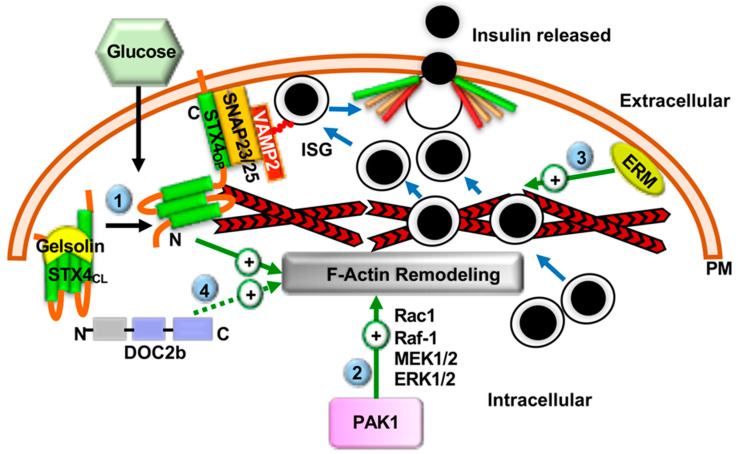
Role of SNAREs and SNARE-associated proteins in F-actin remodeling and GSIS. In the β-cell glucose stimulation dissociates gelsolin from the STX4. This results in the transition of STX4 from its closed (STX4_CL_) to open (STX4_OP_) conformation and F-actin remodeling via direct interaction with the F-actin network (❶). F-actin remodeling increases ISG movement towards PM and SNARE-mediated insulin secretion. Glucose-induced activation of PAK1 facilitates F-actin remodeling and recruitment of ISGs to the PM to support the second phase of insulin release, via activation of Rac1, Raf-1, MEK1/2, and ERK1/2 (❷). Glucose stimulation activates ezrin-radixin-moesin (ERM) proteins and activated ERM translocate to the PM and positively regulates ISG docking via interacting with the F-actin network (❸). Hypothetical positive regulatory role of DOC2b in F-actin remodeling in the β-cell (❹). Blue arrows depict ISG movements, “+” arrows depict positive regulatory roles. F-actin remodeling branches are depicted by (circled numbers).

Prior to SNARE complex assembly and GSIS, filamentous actin (F-actin) remodeling plays an important role in ISG recruitment [80,81,82,83] (Figure 4). For example, while F-actin associates in macromolecular complexes with multiple t-SNAREs and prevents ISG movement towards the PM in unstimulated β-cells [84,85,86], glucose stimulation transiently induces F-actin remodeling and disrupts the t-SNARE: F-actin interaction, permitting ISG access to the t-SNARE docking sites at the PM. DOC2b and STX4, in particular, are noted for their abilities to forge novel interactions with cytoskeletal proteins. In vitro studies, have confirmed that F-actin directly interacts only with STX4, via a unique amino-terminal α-spectrin-like domain within STX4. On the other hand, F-actin interacts indirectly with other SNARE proteins, such as STX1, STX2, STX3, t-SNARE SNAP25, VAMP2, and another v-SNARE VAMP8 [84,86,87,88,89,90].

The F-actin network also acts as a binding platform for additional proteins required for GSIS [84,85,86], such as the Ca^2+^-activated F-actin severing/capping protein gelsolin. Gelsolin directly regulates STX4-mediated GSIS in the β-cell [84]. In absence of Ca^2+^, gelsolin prevents the formation of the SNARE complex via direct binding to STX4. Glucose-stimulated Ca^2+^ influx causes a conformational change that dissociates gelsolin from STX4, thereby facilitating SNARE complex formation and ISG exocytosis [84] (Figure 4). Hence, the gelsolin-STX4 interaction restrains unsolicited insulin release from β-cells in the absence of the appropriate glucose stimulus.

The role of DOC2b in the remodeling of cytoskeletal proteins came from a cancer study using cervical cancer cells showing replenishment of DOC2b, which is otherwise reduced in cancer cell lines, resulting in increased cytoskeletal remodeling and reduced cell migration leading to decreased cancer cell growth [16]. In line with this, both STX4 and DOC2b is known to associate with microtubule-associated Tctex-1 type proteins, reinforcing the regulatory contribution of both of these exocytosis proteins in cytoskeletal remodeling [84,91,92]. Recently, DOC2b was shown to undergo tyrosine-phosphorylation at tyrosine 301, within its functionally indispensable C2B domain following insulin stimulation in the skeletal muscle, resulting in its association with microtubule protein kinesin light chain 1 (KLC1) [93]. KLC1 is a cytoskeletal motor protein with a proposed role in protein/vesicle translocation. The association of DOC2b-KLC1 is critical for its stimulatory role in glucose uptake in the skeletal muscle [93]. However, the precise contribution of DOC2b in cytoskeletal remodeling in the β-cell during insulin exocytosis is not known and calls for future investigation (Figure 4).

The link between β-cell glucose stimulation and F-actin remodeling involves the small Rho family GTPases Cdc42 and Rac1 [94,95,96,97]. Glucose-mediated activation of Cdc42 leads to activation of PAK1, which initiates a signaling cascade via activation of Rac1, Raf-1, MEK1/2, and ERK1/2 to induce F-actin remodeling and recruitment of ISGs to the PM for second-phase GSIS [98,99,100] (Figure 4). Consistent with these studies, PAK1 protein levels are reduced by ~80% in the islets of humans with T2D, compared with non-diabetics, suggesting that deficiency of PAK1 or defects in PAK1 signaling may correlate to T2D susceptibility [98,99,101]. Very recent studies point to roles for the adaptor proteins APPL1 and APPL2 in F-actin remodeling via suppressing the Rac GTPase activating protein 1 (RacGAP1)-mediated inhibition of Rac1 activation [102]. While Rac1 is implicated only in second phase GSIS [103,104], APPL2 regulated both phases of GSIS. This might indicate a second role or binding partner for APPL2 that will need to be identified to reconcile this apparent disconnect. Interestingly, APPL1, which has the same domain organization and high protein sequence homology as APPL2, augments first-phase GSIS via upregulating the mRNA and the protein levels of SNARE proteins STX1, SNAP25, and VAMP2, indicating that these similar APPL isoforms play complementary functional roles to support insulin exocytosis [105]. As the underlying mechanism, APPL1 deficiency attenuated insulin-stimulated Akt activation in both primary pancreatic islets and rat β-cells, whereas adenovirus-mediated expressions of APPL1 rescued GSIS [105]. These results altogether revealed that APPL1 links insulin-stimulated Akt activation to GSIS by upregulating the expression of the core exocytotic machinery proteins in the β-cell [105].

The ERM scaffolding proteins, Ezrin, Radixin, and Moesin, have also been implicated as regulators of F-actin remodeling in GSIS. Each ERM protein contains an F-actin binding domain and a PIP2 binding domain [106,107,108]. All three ERM proteins are expressed in the mouse β-cell, with radixin being the most abundant in mouse islets and β-cells [109]. Glucose stimulation activates the ERM proteins via Ca^2+^-dependent phosphorylation; the activated phosphorylated ERM proteins translocate to the cell periphery where they link PIP2 to F-actin, a step essential for trafficking and docking of ISGs to the β-cell PM [109] (Figure 4). Hence, whereas Cdc42 signaling via PAK1 regulates the initial glucose response that supports F-actin remodeling and second phase GSIS, ERM proteins appear to be involved in the later stages of the signaling cascade. Reduced ERM protein activity has been observed in diabetic ob/ob mouse islets [109], consistent with a model wherein loss of ERM protein function contributes to T2D pathogenesis.

Recent advancements in high spatiotemporal resolution live-cell imaging are likely to facilitate linking these events to provide the first comprehensive understanding of the molecular interactions between proteins on a physiologically relevant timescale underlying the cytoskeletal changes with the coordinated assembly of the exocytosis machinery in secretory cells [110,111,112]. Toward this goal, two-photon fluorescence lifetime imaging of primed SNARE complexes in the β-cell has revealed that SNARE complexes are unassembled in the unstimulated state, and stimulation leads to slow assembly of SNARE complexes [113]. This finding supports prior models wherein DOC2b, Munc18c, and transient F-actin disassembly cause STX4 to take on a conformational state conducive to glucose-stimulated assembly with SNAP25 and VAMP2 for GSIS.

In addition to the above-mentioned proteins and their signaling mechanisms for regulating GSIS, insulin exocytosis from the β-cell is also regulated by secretion-potentiating agents like Glucagon-like-peptide-1 (GLP-1) and cAMP releasing agents [114]. GLP-1 and its analogs have widely been used as therapeutic agents for T2D [115,116]. GLP-1 is an incretin hormone, secreted from the intestinal L-cells in response to glucose and other ingested nutrients and boosts insulin secretion via Glucagon-like-peptide-1 receptor (GLP-1R) in a glucose-dependent manner (reviewed in [117]). Previous studies showed that the insulinotropic effects of GLP-1 depend on the cAMP-Epac2 signaling pathway and is capable of restoring first phase insulin secretory defects in diabetic β-cells [118,119,120]. Interestingly, recent studies point towards the beneficial role of GLP-1 signaling in potentiating insulin secretion under glucotoxic conditions via actin cytoskeleton remodeling [121,122]. As an underlying mechanism, it has been shown that in the INS 832/13 cells (rat clonal beta-cell line) GLP-1 mediated improvement insulin exocytosis under glucotoxic condition is associated with partial restoration of actin dynamics and ISG fusion during both the phases of GSIS, via a preferential involvement of Epac2 signaling in the first phase and protein kinase A (PKA) signaling in the second phase of insulin exocytosis [122].

GLP-1 stimulates PKA-dependent phosphorylation of synaptotagmin-7, a crucial Ca^2+^ sensor affiliated with SNARE-mediated exocytosis, thereby boosting glucose- and Ca^2+^-stimulated insulin secretion [123]. Liraglutide, a GLP-1 analog, was reported to modulate plasma membrane-raft clustering in RIN-m5f β-cells, possibly impacting functionality of raft-embedded SNARE protein isoforms STX1, SNAP25, and VAMP2 [124]. Indeed, earlier studies showed GLP-1 to enhance insulin secretion via mobilizing and docking an increased number of ISGs at the plasma membrane and accelerating sequential ISG—ISG fusions [118]. In line with this, a study using wild type and truncated form of SNAP-25 overexpressing INS-1 cells (rat clonal β-cell line) revealed the requirement of fully functional SNAP-25 in GLP-1 mediated boosting of insulin release, specifically at the level of mobilization of RRP of ISG granules [125]. Additionally, cAMP potentiation via GLP-1 is also known to rescue priming defects caused by Munc13-1 deficiency via Epac and PKA signaling pathways, again confirming the crucial role of GLP-1 signaling in SNARE-mediated insulin release from the β-cell [119]. Interestingly, it has also been reported that VAMP8, a SNARE protein that is known to play a nonessential role in the membrane fusion processes because of functional redundancy contributed by other VAMPs, partially mediates the insulinotrophic effect of GLP-1 [69].

### 2.5. The Ability of SNARE Proteins to Restore β-Cell Function in Diabetes

Human T2D islet β-cells show alterations in the expression levels of SNAREs and SNARE-associated proteins, and rodent models of diabetes show similar changes [20,126,127,128,129,130]. For example, the levels of STX1, STX4, and Munc18a are reduced in T2D human islets compared to non-diabetic islets [20]. In line with this, low DOC2b transcript levels have been reported in the islets of diabetic mice, consistent with a link between loss of DOC2b function and pathogenicity of diabetes [131,132]. It is conceivable that compromised levels of SNARE proteins limit the exocytosis of insulin from β-cells, creating an ISG bottleneck at the most distal step of GSIS. From a therapeutic standpoint, it is important to understand whether the restoration of SNARE proteins is sufficient to restore GSIS. Furthermore, it is important to clarify whether restoring certain SNAREs is sufficient for GSIS. This was addressed initially in the Goto-Kakizaki (GK) rat, a non-obese T2D model characterized by reduced expression of STX1 and SNAP-25 in pancreatic islets [128]. Adenovirus-mediated restoration of STX1 and SNAP25 protein levels was sufficient to improve GSIS, indicating that the SNARE proteins are critical for reversing T2D [128]. Similarly, restoration of STX4 to T2D human islets, which are deficient in STX1, SNAP25, and STX4, was sufficient to restore GSIS to levels seen in age/gender-matched non-diabetic human islets, showing that GSIS can be restored without restoring the levels of all SNARE proteins [20].

Enrichment of individual SNARE accessory proteins can be similarly effective, as DOC2b overexpression in the β-cells of mice prevents the diabetogenic effects of STZ treatment on whole-body glucose tolerance and loss of β-cell mass [78]. Interestingly, while upregulation of STX4 or DOC2b expression in β-cells protects against β-cell damage and dysfunction in diabetogenic mouse models [18,19,78], upregulation of STX1 or Munc18c expression dysregulates GSIS [48,55,133]. As discussed in the previous section, these disparate outcomes are potentially linked to differences in binding partners of these SNAREs and SNARE regulators, which can impact actin cytoskeletal remodeling (e.g., STX4) or interfere with ion channel functions (e.g., STX1). SNARE stoichiometry is also important, since SNARE core complexes are composed in a specific 1:1:1 ratio, and loss/gain of any member of the complex can cause unexpected interactions with other proteins, as was the case with upregulated STX1, which disrupts ion channel function [48]. In summary, these results suggest that very specific isoforms of individual SNAREs can rescue the loss of GSIS but that the balance of SNARE protein levels is critical for maintaining and boosting glucose homeostasis; knowing which ones can be safely overexpressed is key to positive therapeutic outcomes.

## 3. Exocytosis Proteins and β-Cell Protection

Peripheral insulin resistance during pre-T2D places an immense workload on the β-cells to release an extraordinary volume of insulin. To maintain normal glycemic levels, the β-cell mass increases as a compensatory mechanism in response to the insulin resistance induced workload; though, whether the increased mass of β-cells function normally remains in debate [134]. Chronic insulin resistance eventually overwhelms the capacity of the β-cells. When the β-cell can no longer produce enough insulin, glucose homeostasis is disrupted and loss of β-cells ensues primarily via mechanisms such as apoptosis and de-differentiation. Hence, proteins that can protect against β-cell loss are candidates for therapeutic development. Towards this, the ability of exocytosis proteins to protect β-cells from this fate is discussed in this section.

### 3.1. Diabetes-Associated Genes Revealed by Transcriptomic Profiling of β-Cells

**Figure 5:**
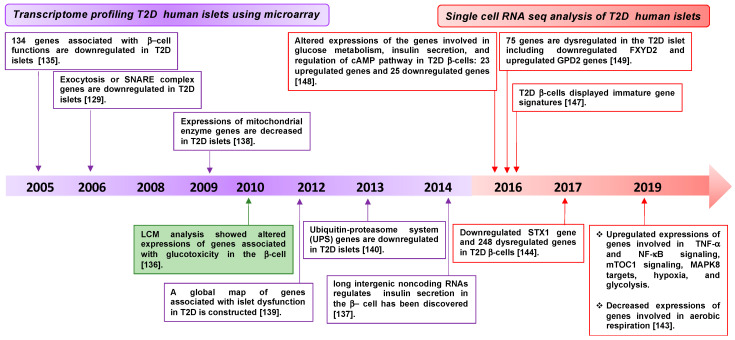
Timeline of human islet transcriptome analysis details from the diabetic and non-diabetic donors. Transcriptome profiling via microarray, GWAS and laser capture (LCM) analyses between 2006 and 2014 elucidated the first associations between human T2D genes and changes attributed to β-cell function. After 2016, analyses transitioned largely to single-cell RNA sequencing (seq), providing β-cell specific changes. [numbers] refer to the list of references.

Transcriptomic profiling has been used to gain novel insight into mechanisms underlying the dysfunction of pancreatic islet β-cells during T2D. Human islet cell transcriptomic analysis of T2D and non-diabetic donors identified multiple genes, such as HNF4α, IR, IRS2, Akt2, and ARNT, as differentially expressed [135]. In addition, microarray analysis of T2D human islet tissue collected using laser capture microdissection (LCM) has revealed downregulation of the genes associated with β-cell function, relative to non-diabetic controls [136]. The candidate genes include those involved in insulin secretion, such as the SNARE and SNARE accessory genes (SNAP-25, VAMP2, STX1, Munc18a, Munc13-1, and synaptophysin). Long intergenic noncoding RNAs and genes involved in metabolic activity and protein homeostasis, such as mitochondrial genes and genes in the ubiquitin-proteasome system, respectively, are also included [129,135,137,138,139,140] (Figure 5 and Table 1).

Given the altered levels of other factors such as STX4 and DOC2b reported in T2D human islets it was surprising that these genes were not detected in the transcriptomic profiling. One possibility for the discrepancy is that unlike the other SNARE genes detected, STX4 and DOC2b are widely expressed across cell types, and that transcriptomics is generally performed using IEQs, or “islet equivalents”, which are known to contain non-islet cells to varying degrees. The contribution of STX4 and DOC2b from non-islet cells in the IEQs would effectively dilute any deficiencies present in the islets. Comparison analyses of STX4 and DOC2b levels in hand-picked T2D human islets vs. IEQs will be required to test this hypothesis.

In addition, β-cell heterogeneity [141] makes it challenging to accurately interpret the whole-islet transcriptome data and may complicate the identification of the precise molecular mechanisms for islet dysfunction in T2D. To overcome this limitation, single-cell RNA sequencing approaches are now used to identify differentially expressed genes and gene groups between non-diabetic and T2D donor islets [142,143,144,145,146,147,148,149] (Figure 5 and Table 1). A single-cell RNA sequencing study identified 248 differentially expressed genes between non-diabetic and T2D donor β-cells, which confirmed a strong correlation between the expression of β-cell-specific genes and T2D. Among the genes decreased by T2D were the insulin and STX1 genes [126,143,144]. In line with these studies, a recent temporal transcriptomic and proteomic analysis of the pancreatic islets procured from T2D rats demonstrated attenuated levels of exocytosis factors like STX1 and STXBP1 [150]. Indeed, one study identified an altered β-cell gene signature in the T2D donor islets. For example, the expression of genes involved in insulin secretion was attenuated, but there was increased expression of hypoxia genes, MAPK8 targets, mTORC1 signaling genes, TNF-α mediated NF-ĸB signaling genes, and immature β-cell gene signatures, indicating de-differentiation [143]. A recent single-cell data highlighted that genes known to be involved in aging are also related to stress signaling in the pancreas [151] (Figure 5 and Table 1). Cumulatively, the results of these transcriptomic studies are consistent with β-cell stress, dysfunction, and dedifferentiation being the key differences between T2D and non-diabetic islets.

### 3.2. Regulation of Exocytosis Proteins by Diabetogenic Stressors

It is well established that genetic and environmental factors, such as obesity and inflammation, accelerate the development of T2D. New insights, as discussed in the following paragraphs, have implicated regulatory cross-talks between exocytosis factors and the diabetogenic risk factors in the process of β-cell disruption during T2D.

#### 3.2.1. Regulation of Exocytosis Proteins and Obesity

The causes of T2D are thought to be chronic exposure to high levels of circulating FFA and glucose. High glucose exposure, in vitro and in vivo, was shown to rapidly reduce the protein levels of the v-SNARE proteins VAMP2 and VAMP3, but not the t-SNARE proteins STX1, SNAP25, or SNAP23, in rodent models [152]. The effects of elevated glucose levels on the other SNARE proteins and the effects of FFA exposure remain to be determined. A recent study showed that overexpression of fatty acid translocase CD36, which is upregulated in obese T2D patients, diminishes SNARE protein expression, and disrupts ISG docking and GSIS in human islet cultures [153]. The authors established a link between overexpression of CD36 and reduced expression of the exocytotic proteins SNAP25, Munc18a, and VAMP2, potentially via a CD36-mediated attenuation of the nPKC-IRS-PI3K/AKT signaling pathway [153]. An antibody to CD36 reversed the dysfunctional GSIS associated with CD36 overexpression, indicating that CD36 is a candidate therapeutic target for restoring SNARE protein levels and GSIS during obesity-associated T2D [153].

#### 3.2.2. Regulation of Exocytosis Proteins and Immune Signaling

Crosstalk between the innate immune system and GSIS was established via detection of an unexpected interaction between the complement system protein CD59 and the SNARE proteins VAMP2 and STX1 in INS-1 β-cells [154]. Otherwise, in non-β-cells, CD59 was known to protect cells from complement-mediated membrane perforation and cell death by translocating to the PM and inhibiting the formation of the membrane attack complex (MAC) [155]. However, in β-cells, the silencing of CD59 decreased GSIS by destabilizing lipid rafts [154]. Furthermore, enzymatic removal of extracellular CD59 did not suppress GSIS, suggesting that an intracellular pool of CD59 is important for regulating β-cell function [154]. This finding indicates that loss of CD59 may both enhance complement-mediated cell killing and directly disrupt GSIS through different mechanisms, making CD59 a potential target for preserving β-cell function and protecting against immune attack. Recently, INS-1 β-cells were shown to contain a cytosolic non-GPI-anchored form of CD59, which facilitates GSIS via interacting with VAMP2 and STX1 [154,156]. Consistent with a role for CD59 as a link between T2D and immunity, another group identified that glycated CD59, which is an inactivated form of CD59 caused by exposure to high blood glucose levels, is elevated in the blood of T2D patients and is decreased by insulin treatment [157]. Thus, CD59 is an intriguing candidate biomarker for T2D as well as a possible therapeutic candidate. However, the precise mechanism linking immunity to GSIS via the SNARE proteins needs further investigation, which may reveal additional candidates as clinical targets.

### 3.3. SNARE Proteins Can Modulate Inflammatory Signals in the β-Cell

Earlier studies have demonstrated a link between low-grade chronic inflammation due to mononuclear cells and T2D [158,159,160,161,162]. Macrophage infiltration has been observed in human T2D pancreatic islets and the islets of diabetes models such as db/db mice and GK rats, as well as pre-T2D models such as C57BL/6 mice fed a high-fat diet. Furthermore, the T2D milieu, which includes elevated FFA plus glucose, drives the production of chemokines, and chemokines expressed by β-cells attract macrophages for infiltration [163,164,165]. Inflammatory factors such as IL-1β, Fas, and NF-κB, and ER stress factors, are important contributors to β-cell dysfunction in T2D. IL-1β and TNFα are the predominant inflammatory cytokines produced by pro-inflammatory M1 macrophages in T2D islets and are known to upregulate stress-related pathways via NF-κB signaling in the islet β-cells [160,166]. Hence, from the above discussion, it is apparent that pancreatic β-cell inflammation positively contributes to the β-cell stress and death in T2D [167]. Mitigating islet inflammation and augmenting β-cell function could be one of the most effective therapeutic strategies to combat complex diseases like diabetes. In line with this, certain SNARE proteins have recently been shown to mitigate islet inflammation, as is discussed below.

**Figure 6:**
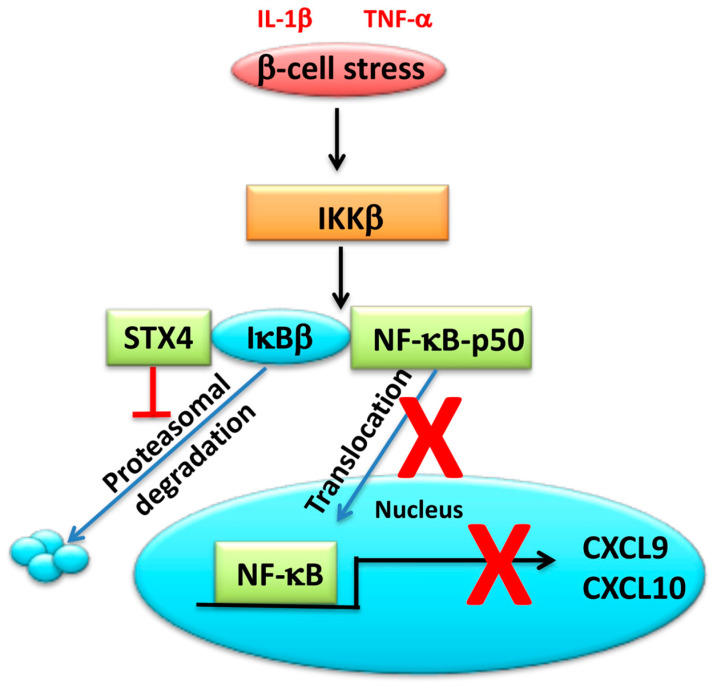
Role of STX4 in modulating inflammatory signals in the β-cell. STX4 binds to IĸBβ and prevents IL-1β and TNFα-induced proteasomal degradation of IĸBβ, thereby reducing p50-NF-ĸB nuclear translocation and suppressing gene expression of T2D-associated chemokine ligands CXCL9 and CXCL10. “T” arrow and red “X” denote inhibitory functions.

The transcription factor NF-κB plays a central role in regulating β-cell inflammation in T2D by inducing many inflammation-related genes, including the characteristic CXCL9 and CXCL10 chemokine ligands of T2D [168,169,170]. Overexpression of STX4 in β-cells was found to not only increase ISG exocytosis but also suppress NF-κB signaling by preventing its translocation to the nuclear compartment [18] (Figure 6). Most recently, STX4 was shown to block NF-κB nuclear translocation by associating with NF-κB inhibitor IκBβ, preventing its degradation and retaining the associated p50-NF-κB in the cytoplasm (Figure 6) [171]. This establishes an unconventional mechanistic cross-talk between exocytosis factors and inflammatory pathway components in β-cells.

STX4 levels were reduced in pro-inflammatory cytokine exposed human islets [18], identifying STX4 itself as a target of cytokines. Work from the cancer field identified STX4 serine 78 as a potential phosphorylation site that targets STX4 for proteasomal degradation; mutation of this serine to alanine resulted in a stabilized form of STX4 [172]. Extrapolating this to the β-cell, the IKKβ subunit, in particular, was implicated as a kinase for STX4, given that an IKKβ inhibitor was able to stabilize the STX4 protein [171]. Furthermore, expression of a Ser78Ala-STX4 mutant suppressed cytokine-induced IκBβ protein degradation, NF-κB nuclear translocation, and CXCL9 expression. Work from the neuronal field points to yet another post-translational modification possibility for STX4 regulation, S-nitrosylation. S-nitrosylation of STX1 at cysteine 145 destabilized its binding with Munc18a to promote synaptic vesicle exocytosis [173]; S-nitrosylation of STX4 at cysteine 141 similarly augments insulin exocytosis in β-cells [174]. These findings highlight the complexity of differential post-transcriptional modifications that can influence the function and resilience of β-cells in the context of β-cell stressors.

In addition to the regulatory role for STX4 in β-cell inflammatory signaling, DOC2b ameliorates β-cell stress during diabetes. Global heterozygous DOC2b^+/–^ KO mice are highly susceptible to STZ treatment, with increased β-cell apoptosis and a smaller β-cell mass compared to their wild type littermates [78]. By contrast, inducible β-cell-specific DOC2b overexpressing mice retain functional β-cell mass following STZ treatment [77,78]. DOC2b enrichment protects β-cells from cytokine-induced apoptosis and ameliorates ER stress, and a truncated portion of the DOC2b protein containing only the two C2 domains is needed to confer this resilience [78]. Post-translational modification of tyrosine 301 within the C2B domain of DOC2b was found to confer resilience to stressed skeletal muscle cells [93], and may function similarly in β-cells. Examination of nonconventional binding interactions, as well as their susceptibilities to stress-induced post-transcriptional/translational modifications, will provide insight into how the SNARE complexes function and how to stabilize them to confer β-cell resilience against diabetogenic stress.

### 3.4. Exocytosis Proteins and Aging

**Figure 7:**
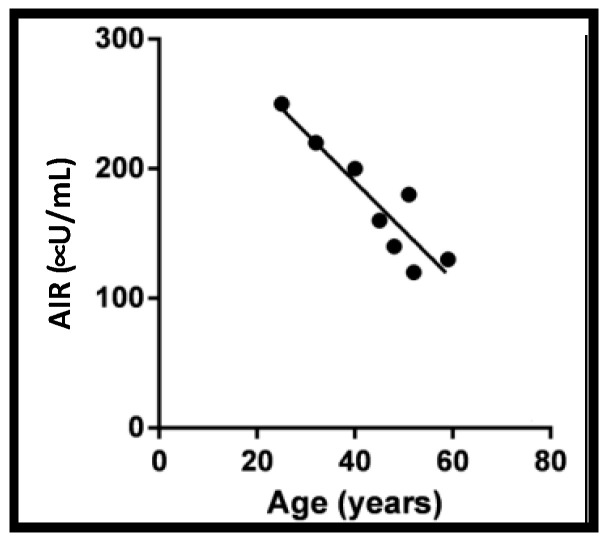
Inverse association between the peak amplitude of first phase GSIS and human islet donor age. (R^2^ = 0.84, *p* = 0.0014). Non-diabetic human donor islets were cultured overnight upon arrival and hand-picked to eliminate non-islet debris. Islets were evaluated by perifusion analyses. First-phase insulin release/acute insulin release (AIR) was quantified in 8 sets of donor islets across a 40-year span of ages.

The risk of developing T2D begins to rise around age 45 and rises considerably after age 65 [175]. Aging is an important risk factor for T2D and several aging mechanisms, such as chronic inflammation, macromolecular dysfunction, and cellular senescence, have also been implicated in the generation of insulin resistance [176]. T2D is also a risk factor for a shortened lifespan [177,178]. Age-associated defects in β-cell GSIS have been demonstrated in both rodents and humans [179,180]. Moreover, islet transplantation outcomes are worse when the islets are from aged donors compared with those from young donors [181], possibly related to diminished peak first-phase GSIS in more aged human islets (Figure 7). Despite a strong correlation between aging and T2D, the mechanism of age-related deterioration in functional β-cell mass is not clear. β-cell mass is controlled by the balance of apoptosis and proliferation. In both rodents and humans, aging limits the regenerative capacity of β-cells, and decreases β-cell proliferation in response to increased metabolic demands [182,183]. High glucose induces apoptosis in human β-cells via inducing the expression of Fas receptor and activating caspases 8 and 3 [184]. β-cell apoptosis is elevated 3–10-fold in T2D compared to non-diabetic individuals, as detected by TUNEL staining [3]. Additionally, cellular senescence could contribute to the functional decline of β-cells during aging. Cellular stressors increase with age, leading to the accumulation of senescent β-cells [185]. The subpopulation of senescent cells was increased in human islets by age and this portion was enriched in T2D human islets compared to age-matched non-diabetic donors [186]. Senescent β-cells are abnormally large and are characterized by upregulated expression of p16^INK4a^ and the anti-apoptotic molecule Bcl2, along with senescence-associated β-galactosidase, as well as the senescence-associated secretory phenotype (SASP) such as IL-6, IL-8, MCP-1 (monocyte chemoattractant protein-1), PAI-1 (plasminogen-activated inhibitor-1), and IGF1R [179,186,187,188], although marker expression is notably heterogeneous. Consistent with the coupling of T2D with early aging, induction of insulin resistance increases the expression of aging markers [179]. It has been proposed that β-cell senescence during aging impairs the expression of genes relevant to β-cell identity and cellular function [179,186]. Moreover, senescence-associated cytokines could trigger an inflammatory response, exacerbating β-cell dysfunction, and demise. Hence, decreasing the senescent population in β-cells is thought to improve β-cells function and glucose regulation [186]. However, contradicting this widely accepted view, β-cell-specific overexpression of p16^INK4a^ in mice improved GSIS, rather than impaired GSIS [189]. As a result, while it is clear that p16^INK4a^ exerts complex influences on β-cells, the underly- ing mechanistic details must await further study.

STX4 protein, a key regulator of GSIS and found in reduced quantities in T2D human islets, is similarly reduced in the pancreata of aged mice [19]. Global STX4 overexpression (2–5-fold STX4 overexpression detected in skeletal muscle, adipose, and pancreas) extended lifespan by ~35%; underlying this was the retention of youthful insulin sensitivity and GSIS capacity, even in the face of diet-induced obesity stress, indicating an anti-aging role for STX4 beyond conventional exocytosis function [19]. Mechanistically, although classical aging-related genes such as Sirt1, mTOR, and aging-related inflammatory factors TNFα or IL-6 were unchanged, phosphorylated Foxo1 was significantly decreased in the pancreata of the aged STX4 transgenic mice [19]. Evaluation of senescence in islet β-cells from the long-lived STX4 mice will be an important step forward in interrogating the mechanistic link among exocytosis proteins, T2D, and aging. It has been reported that activation of NF-κB signaling in normal somatic cells enhances aging and accelerates senescence via upregulation of SASP associated genes and downregulation of genes for cell cycle progression [190]. Given the recently discovered role of STX4 in attenuating IĸBβ degradation and thereby blunting NF-κB signaling [18,171], it is conceivable that STX4 may impact β-cell senescence and proteostasis via an IĸBβ-NF-κB-dependent mechanism. In summary, exciting recent discoveries are pointing towards a previously unexplored, unconventional function for classical exocytosis proteins, establishing them as mediators of healthy aging and resistance to T2D.

## 4. Future Perspectives

The primary aim for T2D treatment is to attain and maintain whole-body glucose homeostasis, via boosting pancreatic β-cell function and mitigating the stressful workload impinged by persistent peripheral insulin resistance. Several SNARE and SNARE-associated proteins are promising therapeutic candidates, including STX4 and DOC2b. The mRNA and protein levels of both these proteins are reduced in T2D islet β-cells, suggesting that their deficiency may contribute to the pathogenesis of T2D. However, while STX4 nor DOC2b has been reported as T2D susceptibility genes per se, the STX4 gene associates with BMI (http://type2diabetesgenetics-old.org/). Intriguingly, STX4 was identified in an in silico phenome–interactome analysis, a method that prioritized candidates according to their physical interactions at the protein level with other proteins involved in type 1 diabetes. STX4 was in the top 10 in a list of genes predicted to be likely disease genes in T1D, including the insulin (INS) gene. Further development of these proteins as drug targets will reveal whether they can rescue β-cell dysfunction in the clinical setting.

Based on the promising beneficial contribution of extra STX4 and DOC2b in regulating whole-body glucose homeostasis and the reduced level of these two key exocytosis factors in T2D β-cell, it is imperative to explore the ways to induce their protein levels in the β-cell. The major technical challenge for the induction of targeted protein expression in the β-cell is the complex 3D structure of islets. Several approaches are now being tested including small activating RNA (saRNA) mediated induction of endogenous proteins, use of adeno-associated virus vectors (AAV) as a delivery system [191,192]. Another alternative therapeutic approach is the transplantation of pancreatic islets harboring enhanced levels of STX4 or DOC2b into T2D patients [193]. Future studies are required to identify and optimize ways to enrich levels of these two key exocytosis proteins in the β-cell. It is worth mentioning here that at this time other drugs that modulate insulin exocytosis to protect β-cells are upstream of SNARES. For example, Sulfonylureas (chlorpropamide, glipizide, etc.) increase insulin secretion via binding to ATP-dependent K^+^ channel [1,194]; GLP-1 agonists (liraglutide, exenatide) boost insulin secretion via binding to GLIP-1 receptor in the β-cell [1,195]; and additionally, inhibitors of Thioredoxin-interacting protein (TXNIP), promote insulin production and GLP-1 signaling via regulation of microRNA, are emerging as a potential treatment option for diabetes [196,197]. However, since all of these above-mentioned drugs work upstream of SNAREs, there may be effects beyond the distal steps of insulin exocytosis, again implying the significance of developing drugs for specific modulations of SNARE and SNARE regulatory proteins in the β-cell.

It is important to mention here that not all SNARE proteins are candidates for therapeutic development, as extra STX1 in the β-cell interferes with Ca^2+^ channel gating and impairs GSIS. Also, extra STX2 would be detrimental in T2D β-cells because of its inhibitory function. These data again remind us of the essential requirement for the proper stoichiometry during SNARE complex assembly and successful GSIS. It is imperative to suggest that the exocytosis factors that are conferring beneficial function when overexpressed are possibly doing so via indirectly influencing specific signaling pathways, such as STX4 mediated inhibition of NF-κB nuclear translocation, or possibly via direct protein-protein interactions. One such example is the direct interaction of STX4 with key proteins of the actin cytoskeleton, including F-actin itself, in the β-cell. These findings altogether suggest that our growing understanding of exocytosis proteins is opening up new avenues for T2D treatment.

## Figures and Tables

**Table 1:** Transcriptomic studies of β-cell dysfunction in T2D.

Material	Technique	Genes Identified	Reference
Human T2D islets	Microarray and qRT-PCR	Dysregulation of 370 genes (134 genes downregulated associated with β-cell function [e.g., HNF4a, IR, IRS2, AKT2, ARNT]).	[135]
Human T2D islets	Microarray and qRT-PCR	Decreased expression of exocytotic SNARE complex proteins (STX1A, SNAP25, VAMP2, Munc18-1, Munc13-1, Synaptophysin).	[129]
Human T2D islets	Microarray and qRT-PCR	Decreased expression of metabolic enzymes (mitochondrial enzymes).	[138]
Human T2D pancreas	Microarray of islets captured by LCM	Increased expression of genes associated with glucotoxicity. Decreased expression of exocytotic protein, SNAP25.	[136]
Human T2D islets	Microarray, qRT-PCR and SNP array	A global map of genes associated with islet dysfunction. Decreased expression of genes related to insulin secretion (KCNJ11, WFS1, SLCA2A, JAZF1, G6PC2).	[139]
Human T2D islets	Microarray, qRT-PCR	Downregulation of ubiquitin-proteasome system genes.	[140]
Human T2D islets	RNA-seq	Decreased expression of long intergenic noncoding RNAs (LOC283177) which positively associate with insulin exocytosis.	[137]
Human T2D islets	Single-cell RNA-seq	Dysregulation of 48 genes which are involved in sensing and metabolism of glucose and regulating cAMP pathways, correlated with insulin secretion.	[148]
Human T2D islets	Single-cell RNA-seq	Dysregulation of 75 genes; downregulation of FXYD2 (unlike the findings in ref 4) and upregulation of GPD2.	[149]
Human T2D islets	Single-cell RNA-seq	A more immature gene signature found in T2D β-cells	[147]
Human T2D islets	Single-cell RNA-seq	Downregulation of 248 genes in T2D β-cells, including STX1A.	[144]
GK T2D rat	RNA-seq and TMT-based proteomics	Downregulation of STX1A and STXBP1.	[150]
Human T2D islets	Single-cell RNA-seq	T2D β-cells with increased TNF-α signaling via NF-κB, MAPK8 targets, mTOC1 signaling, hypoxia, glycolysis, proteasome pathway and decreased aerobic cellular respiration compared to non-diabetic β-cells.	[143]

GK, Goto-Kakizaki; LCM, laser capture microdissection; RNA-seq, RNA sequencing; qRT-PCR, quantitative reverse transcriptase PCR; T2D, type 2 diabetes.
